# A model to calculate cardiac output in hemodialysis patients by thermodilution

**DOI:** 10.1186/1742-4682-9-24

**Published:** 2012-06-21

**Authors:** Ahmed Alayoud, Kawtar Hassani, Mohammed Benyahia

**Affiliations:** 1 Service of Nephrology, Hemodialysis and Kidney Transplantation, Military Hospital of Instruction, Mohammed V, Rabat, 10000, Morocco

**Keywords:** Cardiac output, Dialysis, Thermodilution, Vascular access flow, Recirculation

## Abstract

The Blood Temperature Monitor module (BTM) is used to measure recirculation by thermodilution in dialysis. Numerous studies have confirmed its interest in the measuring of the vascular access flow. In this letter we describe a model to calculate cardiac output in dialysis by the BTM.

## 

The Blood Temperature Monitor module (BTM) is used to measure recirculation by thermodilution in dialysis. Numerous studies have confirmed its interest in the measuring of the vascular access flow [1-3]. In this letter we describe a model to calculate cardiac output in dialysis by the BTM.

The model is based on two principles:

1. The measurement of recirculation rate by the BTM is based on the Fick principle by changing dialysate temperature, which will change the venous blood temperature returning to the patient. A dilution method described and validated by Schneditz et al, using the recirculation values obtained with the haemodialysis (HD) lines in the normal (Rnl) and reverse positions (Rinv), is used to separate the central cardiopulmonary component of recirculation (CPR) from the recirculation fraction and to calculate vascular access flow [3].

2. Removal of solute from the systemic tissue compartment is equal to the removal of solute from the vascular access.

The removal of solute [j = —V x (dCven/dt)] from the systemic tissue compartment (V = urea distribution volume) is the product of the flow through the systemic tissue compartment (Qv) multiplied by the concentration difference of the fluid leaving (Cven) and entering (Cart) the tissue compartment [4]:

(1) $$\text{j }=-\text{V}\frac{dCven}{dt}=\left(Cven-Cart\right)\times Qv$$

The systemic (Qv) and the access (Qa) flows mix in the heart. When solute is cleared from the access flow during HD, this mixing reduces the solute concentration of the mixed arterial blood. Since Cart is reduced, the concentration gradient which can be built up between the blood and the dialysate and therefore the concentration driving force to remove solute from the blood is also reduced.

The amount of transfer j is equal to the removal of solute from the vascular access which is determined by Access clearance (Kac) multiplied by mixed arterial concentration (Cart) [4]. Access clearance (Kac) is dialyzer clearance (Kd) corrected for 'short loop' recirculation at the access site and, therefore, may be less than (Kd).

(Kac) can be measured by ionic dialysance.

(2) $$j=-V.\frac{dCven}{dt}=Kac.Cart$$

Combination of equation (1) with (2) yields:

(3) $$Cven=\left(1+\frac{Kac}{Qv}\right).Cart$$

The recirculation rate (R) may be calcultaed according the following equation:

(4) $$\text{R}=\frac{\left(Cven-Cart\right)}{\left(Cven-Css\right)}$$

(Css) is the concentration leaving the dialyzer:

(5) $$Css=\left(1-\frac{Kd}{Qb}\right).Cart$$

(Kd) is dialyzer clearance which takes no account of the recirculation, and can be calculated using access clearance (Kac) corrected for total measured recirculation and blood flow (Qb) [5]:

(6) $$Kd=Kac.\frac{1-R}{1-R.\left(1+\frac{Kac}{Qb}\right)}$$

From equations 3 to 6 we deduce;

(7) $$Qv=\frac{\left(1-R(1\frac{Kac}{Qb}\right)}{R}\cdot Qb$$

Cardiac output (CO) is the sum of systemic flow (Qv) and access flow (Qa) [4]:

(8) CO=Qv+Qa

Qa is calculated from both recirculation values, obtained with the haemodialysis lines in the normal (Rnl) and reverse positions (Rinv) [3]. The measurement process starts from the production of a « temperature bolus » secondary to the self-limited decrease in the temperature of the dialysis fluid. This thermal decrease is initially sensed by the temperature sensor of the venous line, and after traveling through the cardiopulmonary circulation of the patient, returns already reduced toward the dialyzer and is felt by the temperature sensor of the arterial line. Quantification of the last « arterial temperature bolus » in relation to the « venous temperature bolus » initially generated allows for calculating the recirculation percentage with the HD lines in a normal configuration. The same procedure is repeated with HD lines in a reverse configuration. Qa is calculated from both recirculation values using the following formula without considering the ultrafiltration:

(9) $$\text{Qa}=\frac{\left(1-\text{Rinv})(1-\text{Rnl}\right)}{\left(\text{Rinv}-\text{Rnl}\right)}\text{.Qb}$$

Equation 7 became in the normal position of the haemodialysis lines:

(10) $$Qv=\frac{\left(1-Rnl.(1+\frac{Kac}{Qb}\right)}{Rnl}\cdot Qb$$

Thus, CO can be rewritten in terms of familiar, measurable variables:

(11) $$CO=Qb.\left(\frac{\left(1-Rinv\right).\left(1-Rnl\right)}{\left(Rinv-Rnl\right)}+\left(\frac{1-Rnl.\left(1+\frac{Kac}{Qb}\right)}{Rnl}\right)\right)$$

So cardiac output can be measured using the blood temperature sensor, BTM, incorporated into the dialysis machine according to blood flow, BTM recirculation with reverse and normal placement of blood lines, and ionic dialysance. However the use of the BTM for the measurement of cardiac output is not yet validated, despite an approach for calculating CO by this module has already been advanced in 1999 by Schneditz [3]. This approach is different from that described in our model because it doesn’t take into account the access clearance (Kac). Thus by comparing the two approaches we found that Schneider has neglected the ratio Kac/CO.

In summary, like transonic system, the BTM thermodilution can be used in measurement of cardiac output in haemodialysis patient without additional cost, and therefore could allow tracking of cardiac function in this patient cohort. However a clinical validation of this model by comparison with other reference methods will be needed.

## Competing interest

The authors declare that they have no competing interests

## Authors’ contributions

AA has defined the research theme, developed the model and wrote the paper. KH has been involved in revising the manuscript. MB has given final approval of the version to be published. All authors read and approved the final manuscript.
